# BID and the α-bisabolol-triggered cell death program: converging on mitochondria and lysosomes

**DOI:** 10.1038/s41419-019-2126-8

**Published:** 2019-11-26

**Authors:** Antonella Rigo, Isacco Ferrarini, Erika Lorenzetto, Elena Darra, Irene Liparulo, Christian Bergamini, Cinzia Sissa, Elisabetta Cavalieri, Fabrizio Vinante

**Affiliations:** 1 Department of Medicine, Section of Hematology, Cancer Research and Cell Biology Laboratory, University of Verona, Verona, Italy; 2 Technology Platform Center, University of Verona, Verona, Italy; 3 Department of Medicine, Section of Geriatric Medicine, University of Verona, Verona, Italy; 4 Department of Pharmacy and Biotechnology, FABIT, University of Bologna, Bologna, Italy; 5 Immunohematology, Azienda Ospedaliera Mantova, Mantova, Italy; 6 Department of Life and Reproduction Sciences, Section of Biochemistry, University of Verona, Verona, Italy

**Keywords:** Cancer therapy, Preclinical research

## Abstract

α-Bisabolol (BSB) is a plant-derived sesquiterpene alcohol able to trigger regulated cell death in transformed cells, while deprived of the general toxicity in several mouse models. Here, we investigated the involvement of lysosomal and mitochondrial compartments in the cytotoxic effects of BSB, with a specific focus on the BH3-only activator protein BID. We found that BSB particularly accumulated in cancer cell lines, displaying a higher amount of lipid rafts as compared to normal blood cells. By means of western blotting and microscopy techniques, we documented rapid BSB-induced BID translocation to lysosomes and mitochondria, both of them becoming dysfunctional. Lysosomal membranes were permeabilized, thus blocking the cytoprotective autophagic flux and provoking cathepsin B leakage into the cytosol. Multiple flow cytometry-based experiments demonstrated the loss of mitochondrial membrane potential due to pore formation across the lipid bilayer. These parallel events converged on neoplastic cell death, an outcome significantly prevented by BID knockdown. Therefore, BSB promoted BID redistribution to the cell death executioner organelles, which in turn activated anti-autophagic and proapoptotic mechanisms. This is an example of how xenohormesis can be exploited to modulate basic cellular programs in cancer.

## Introduction

α-Bisabolol (BSB) is a vegetal highly lipophilic sesquiterpene taking part in plant immune defense against microbial insults^1^. Similarly to other plant metabolites, it also succeeds in interacting with diverse intracellular pathways of normal mammalian cells^2^, which usually mount anti-inflammatory and proresolving responses under BSB^3,4^.

In addition, several preclinical studies from our group and others revealed that BSB exerts pleiotropic effects against a wide range of transformed cells, eventually leading to bioenergy dissipation, membrane permeabilization, and regulated cell death (RCD)^5–11^. Because of its low toxicity at the therapeutic dosages, easy administration route and opportunity for synergism^5,11^, BSB has already been tested in animal models^11^ and proposed as a novel antineoplastic agent for treating acute lymphoblastic leukemia^5,8^, chronic myeloid and lymphocytic leukemias^5,7^, malignant gliomas^9^, and pancreatic carcinomas^11^.

However, the sequence of intracellular events triggered by BSB and leading to apoptotic cell death is still lacking clear distinction of elements. Lipid rafts, specialized membrane domains that compartmentalize either prosurvival or proapoptotic signaling molecules^12^, have been reported to directly interact with BSB, allowing it to be absorbed into the plasma membrane^10^. Once entered the cell, a series of injuries involving mitochondria and lysosomes seems to drive the cytolytic program activated by BSB^6^. In chronic lymphocytic leukemia cells, for example, it promoted the opening of mitochondrial membrane pores and destabilized the lysosomal compartment, eventually favoring the collapse of the cell-rescuing autophagic apparatus^7^.

Molecular interactions priming the death cascade are even less clear. The proapoptotic, BH3-only protein BID has been hypothesized to orchestrate the proximal response to BSB, based on some basic observations: it can be recruited into lipid rafts by some apoptosis-inducing agents^13^; it functionally links extrinsic and intrinsic apoptotic pathways, both ending with mitochondrial membrane depolarization and caspase activation^14^; and it is involved in the lipid exchange and trafficking from plasma membranes to intracellular ones^15^. Proceeding on this track, we previously demonstrated that BID moved into flotillin-rich membrane regions upon BSB treatment and, at least in cell-free conditions, it interacts with BSB through its methionine 194, which is part of the hydrophobic groove present on its surface^10^. Therefore, the core proapoptotic protein BID might be considered as a candidate cytoplasmic receptor for BSB, both of them appearing colocalized in lipid rafts.

By using HeLa and Jurkat cells as in vitro cancer models, here we demonstrate that BID is rapidly mobilized upon BSB treatment and its presence is required for full cytotoxicity to occur. Therefore, we present the main cellular events triggered by BSB, involving lysosomal and mitochondrial disruption and ending with neoplastic cell death.

## Materials and methods

### Cells

Normal PBMC were isolated from healthy donors. HeLa (cervical cancer), Jurkat (acute T cell leukemia), CML-T1, K562, MEG-01, and LAMA-84 cell lines (blast crisis of chronic myeloid leukemia) were purchased from DSMZ (Braunschweig, DE). Cell lines were authenticated routinely and were mycoplasma free. Cells were cultured in RPMI-1640 (Invitrogen, Carlsbad, CA), supplemented with 10% heat-inactivated fetal bovine serum (Invitrogen), 50 U/mL streptomycin and 50 U/mL penicillin (complete medium, CM), and maintained at 37 °C in 5% CO_2_.

### Lipid raft component quantification

The main components of lipid rafts (GM1, flotillin-1, and cholesterol) were quantified. (1) GM1: Cells were incubated at 4 °C for 10 min with Alexa Fluor 488 cholera toxin subunit B (CT-B, Invitrogen), which specifically binds to the pentasaccharide chain of GM1. Cells were then washed three times with phosphate buffer solution (PBS) and analyzed by flow cytometry (FACSCalibur or FACSCantoII, Becton Dickinson, San Jose, CA). (2) Flotillin-1: Cells were fixed, permeabilized by a commercial kit (eBiosciences, San Diego, CA), and incubated with an anti-Flotillin-1-Cy3 antibody (Sigma-Aldrich, no. F8931, St. Louis, MO) for 1 h at 4 °C. After washing, samples were analyzed by flow cytometry and fluorescence microscopy. GM1 and flotillin-1 data were expressed in arbitrary units as the ratio between median fluorescence intensity of stained cells (subtracted of that of unstained ones) and median forward scatter (as a surrogate of cell dimension) multiplied by 10^4^. (3) Cholesterol: The cholesterol content was determined as previously described^16^, with minor modifications. The oxidized cholesterol was separated on a Kinetex C18 column (100 × 4.6 mm, 2.6 μm; Phenomenex, Torrance, CA), using a mobile phase consisting in 1:99 acetic acid/methanol (v/v) at a flow rate of 0.4 ml/min with an Agilent 1100 HPLC system. Absorbance at *λ* = 240 nm was monitored by a photodiode array detector.

### BSB extraction

Cells were incubated with 40 μM BSB (Sigma-Aldrich) for 24 h. Then they were harvested, washed twice with PBS, and subcellular fractions were obtained according to Imai et al.^17^. BSB in subcellular fractions was quantified by HPLC following Sao Pedro et al.^18^. All chromatographic runs were performed with a Waters 510 HPLC system, Kinetex C18 column (100 × 4.6 mm, 2.6 µm; Phenomenex) and Waters 996 Photodiode Array detector. Calibration curves for quantification were generated using freshly prepared standard solutions at BSB ranging from 0.2 to 5 nmol.

### Cell cycle

Jurkat cells were treated with either vehicle (dimethyl sulfoxide, DMSO; Sigma-Aldrich) or 20, 40, 80 µM BSB (representing the calculated soluble fraction in the assay as reported elsewhere^5^, dissolved in DMSO 1:40) for 24 h and incubated with 10 μM Vybrant DyeCycle orange reagent (Life Technologies, Carlsabad, CA) for 15 min and with 1 μM Sytox Blue dead Cell reagent (Life Technologies) for additionally 15 min to exclude dead cells from cytometric analysis, as previously reported^19^.

### Apoptosis assay

Jurkat cells were treated for 5 h with 80 μM BSB, washed with PBS, and incubated with Annexin-V-FITC (Miltenyi Biotec, Bergisch Gladbach, DE) for 15 min. TO-PRO-3 (Invitrogen) was added immediately before the flow cytometry acquisition.

### Cytosolic and mitochondrial fraction preparation, and western blot

A total of 5 × 10^6^ HeLa cells were treated with 80 μM BSB for 2, 3, and 5 h. Treatment with 100 μM etoposide (Sandoz, Origgio, IT) for 24 h was used as positive control for BID cleavage. After washing, cells were suspended in 10 mM NaCl, 1.5 mM MgCl_2_, 10 mM Tris-HCl, pH 7.5, 1 mM sodium orthovanadate, and complete EDTA-free protease inhibitor cocktail (Boehringer, Mannheim, DE). Cells were then chilled on ice for 10 min and gently lysed by adding 0.3% NP-40. In order to restore an isotonic environment, a solution containing 525 mM mannitol, 175 mM sucrose, 12.5 mM Tris-HCl, pH 7.5, 2.5 mM EDTA, and protease inhibitor cocktail was added. Lysates were first centrifuged at 600 × *g* at 4 °C, in order to remove nuclei, and then the supernatants were centrifugated at 17,000 × *g* for 30 min at 4 °C. The obtained supernatants were collected and used as the cytosolic fraction. The pellets, which contained mitochondria, were washed once with the same buffer and then were resuspended in the sample buffer. The cytosolic and the mitochondrial fractions were separated on a 15% sodium dodecyl sulfate–polyacrylamide gel electrophoresis (SDS–PAGE) and probed using a rabbit polyclonal IgG antibody to BID (Cell Signaling Technology, no. 2002, Danvers, MA). Then, the membrane with the cytosolic and mitochondrial fractions was probed with a rabbit polyclonal IgG antibody to α-tubulin (Cell Signaling Technology, no. 2144) and with a mouse monoclonal IgG antibody to Hsp60 (Cell Signaling Technology, no. 12165).

For the study of PARP, Jurkat cells were homogenized at 4 °C in Lysis Buffer (Thermo Scientific, Rockford, IL) plus protease inhibitor cocktail (Sigma-Aldrich). Aliquots of whole-cell lysates (40 μg total protein/lane) were loaded on SDS–polyacrylamide gels and the resolved proteins were electroblotted onto a nitrocellulose membrane. Membranes were then incubated with anti-PARP antibody (Zymed Laboratories, no. 333100, South San Francisco, CA) and with anti-GAPDH antibody (Cell Signaling Technology, no. 2118). The membranes were then washed and incubated with a VeriBlot-HRP secondary antibody (Abcam, no. AB131366, Cambridge, UK). Development was made by enhanced chemiluminescent plus detection reagents (Thermo Scientific).

### BID transfection

HeLa cells (5 × 10^4^ cells/mL) were seeded in chamber slides Lab-Tek II (Thermo Fisher Scientific, Waltham, MA) and let to adhere for 24 h. Then they were transfected for 48–72 h by Fugene HD (Promega, Madison, WI) with 0.5 μg pDsRed2-BID expression vector (Clonotech, Mountain View, CA). They were stained with MitoTracker Deep Red FM or MitoTracker Green FM (150 nM, Invitrogen) and LysoTracker Green DND-26 (50 nM, Invitrogen) for 30 min, 80 μM BSB added, and monitored for 3 h by time-lapse imaging confocal microscopy (Leica TCS-SP5 equipped with an incubator with temperature and CO_2_ controllers, Wetzlar, DE). Images, captured every 10 min, and movies were elaborated by using LAS-AF and Imaris software.

### BID silencing

HeLa cells were cultured in 96-well plates and transfected by Lipofectamine RNAiMAX Reagent (Thermo Fisher Scientific) with a mix of two specific BID-siRNA (small interfering RNA; 1.2 + 1.2 pM, Silencer selected validated siRNA; Invitrogen) or control-siRNA for 48 h. Then they were exposed for 96 h to 10, 20, 30, 40, and 80 μM BSB. Silencing was checked by western blotting. At the end of the culture, viability was measured by MTT (Sigma-Aldrich) incorporation, as previously described^20^. Viability was expressed as the ratio between the number of cells treated with BSB and the number of cells treated with the vehicle alone in each condition.

### Detection of autophagic flux

Jurkat cells were treated with vehicle or 80 μM BSB for 16 h. They were harvested, washed in cold PBS, and resuspended in Lysis Buffer (Thermo Fisher Scientific) plus protease inhibitor cocktail (Sigma-Aldrich). Whole-cell lysates were separated on a 12% SDS–PAGE and analyzed by immunoblotting as described above.

LC3: In order to inhibit the degradation of autophagic cargo, Pepstatin A (Sigma-Aldrich, 10 μg/mL) and E-64D (Sigma-Aldrich, 10 μg/mL) were added to the media. Membranes were tested with anti-LC3 (Cell Signaling Technology, no. 12741) and anti-GAPDH antibodies (Cell Signaling Technology, no. 2118).

Beclin 1: Membranes were tested with anti-Beclin 1 (Cell Signaling Technology, no. 3495) and anti-GAPDH antibodies. Control and BID-knockdown Hela cells were also tested.

### RNA isolation and reverse transcription quantitative real‐time polymerase chain reaction

Total cellular RNA was purified by EuroGold Trifast kit (Euroclone, Pero, IT) from HeLa and Jurkat cells treated with either vehicle or 80 μM BSB for 6 and 16 h. Templates were generated from 1 μg of RNA by the High Capacity cDNA Reverse Transcription kit (Applied Biosystems, Foster City, CA). Beclin 1 (TaqMan assay ID Hs1007018_m1, Thermo Fisher Scientific) and GAPDH internal control (Hs99999905_m1, Thermo Fisher Scientific) were coamplified with the Taqman Fast Advanced Master Mix (Thermo Fisher Scientific) in technical duplicate using the 7500 Real time PCR System (Applied Biosystems). The thermal cycling conditions were as follows: 50 °C for 2 min, 95 °C for 2 min, followed by 40 cycles of denaturing at 95 °C for 3 s, and annealing at 60 °C for 30 s. The relative expression levels of Beclin 1 were determined by the 2^−ΔΔCt^ method. Data are expressed as fold change.

### Lysosome damage assays

(1) LTG-uptake assay: Jurkat and BID-knockdown HeLa cells were incubated with 80 μM BSB for 5 h. Then they were washed and stained at 37 °C for 1 h with 75 nM of the acidotropic dye LTG (Molecular Probes, Eugene, OR), spotted onto a slide (HeLa were detached with trypsin), mounted with a coverslip, and immediately recorded by confocal microscopy. (2) AO-relocation assay: Jurkat cells were stained with 0.5 μg/mL AO (Molecular Probes) at 37 °C for 15 min, washed twice, treated with 80 μM BSB for 5 h, and evaluated by flow cytometry (FL-1 channel). An aliquot of each sample was spotted onto a slide, mounted with a coverslip, and immediately recorded by confocal microscopy. (3) AO-uptake assay: Jurkat cells were incubated with 80 μM BSB for 5 h and stained with 0.5 μg/mL AO for 15 min at 37 °C, washed twice and analyzed by flow cytometry (FL-3 channel). An aliquot of each sample was spotted onto a slide, mounted with a coverslip, and immediately recorded by confocal microscopy. (4) Cathepsin B immunofluorescence staining: Jurkat cells were grown on slides until semiconfluence, exposed to 80 μM BSB for 5 h, and treated as previously described^7^. Briefly, after fixing they were incubated with a rabbit monoclonal antibody to human cathepsin B (Abcam, no. AB125067), developed with a goat anti-rabbit Alexa Fluor 568-conjugated antibody (Invitrogen, no. A11011), counterstained with DAPI, and imaged by an Axio Observer inverted microscope (Zeiss, Gottingen, DE).

### Mitochondrial damage assays

(1) ΔΨ_m_: As previously described^8^, Jurkat cells were resuspended in CM and treated with 40 μM BSB for 3 and 5 h at 37 °C. Then, they were washed with prewarmed CM, loaded with 4 μM JC-1 (5,5′,6,6′-tetra-chloro-1,1′,3,3′-tetra-ethyl-benz-imidazolyl-carbo-cyanine iodide, Molecular Probes) for 30 min, then washed twice with PBS. Aliquots of each sample were resuspended in PBS and analyzed by flow cytometry or spotted onto a slide, and recorded by an Axio Observer inverted microscope. Visualization of JC-1 monomers and JC-1 aggregates was done using filter sets for fluorescein and rhodamine dyes, respectively. Image analysis was performed using Axiovision 3 software. Control and BID-knockdown Hela cells were also tested. (2) ΔΔΨ_m_ measurement: Cells treated with 20, 40, 80 μM BSB for 5 h were incubated with 100 nM TMRM for 30 min at 37 °C. Then they were washed with PBS and subjected to flow cytometry. (3) mPTP: Cells were treated with 40 μM BSB for 5 h. Then, they were washed, resuspended in HBSS/Ca^2+^, loaded with 10 nM calcein AM with or without 400 μM CoCl_2_ for 15 min at 37 °C (MitoProbe Transition Pore assay kit, Invitrogen), and analyzed by flow cytometry. An aliquot of each sample was spotted onto a slide, mounted with a coverslip, and immediately recorded by confocal microscopy.

(4) Mitochondrial morphology: HeLa cells were plated in ibiTreat m-Slide 15 Well (Ibidi, Gräfelfing, DE) at a density of 3 × 10^4^/well with 50 µl CM, and incubated overnight at 37 °C in a humidified atmosphere and 5% CO_2_. Afterward, cells were transfected with a plasmid carrying the GFP targeted to mitochondria (CellLight Mitochondria-GFP, BacMam 2.0; Thermo Fisher Scientific) and exposed to 80 µM BSB or vehicle for 24 h. Mitochondrial network morphology was assessed by live-cell imaging using Nikon C1si confocal microscope (Tokyo, JP). The aspect ratio was employed as a mitochondrial morphometric parameter, according to Marchi et al.^21^ Image J standard tools were used to analyze images.

### Statistics

Continuous variables were presented as the mean ± s.d., unless otherwise specified. When appropriate, data were summarized by percentages. Mann–Whitney *U* test was used to analyze the difference between means. Kruskall–Wallis test (or Friedman test for matched data) followed by Dunn’s multiple comparisons test were used to analyze the differences between multiple means. Data had normal distribution. Presented data meet the assumptions of the tests. Differences were considered as significant for *p* < 0.05. Analyses were performed using the statistical software Stata 12.1.

## Results

### Lipid raft components and BSB uptake

**Fig. 1:**
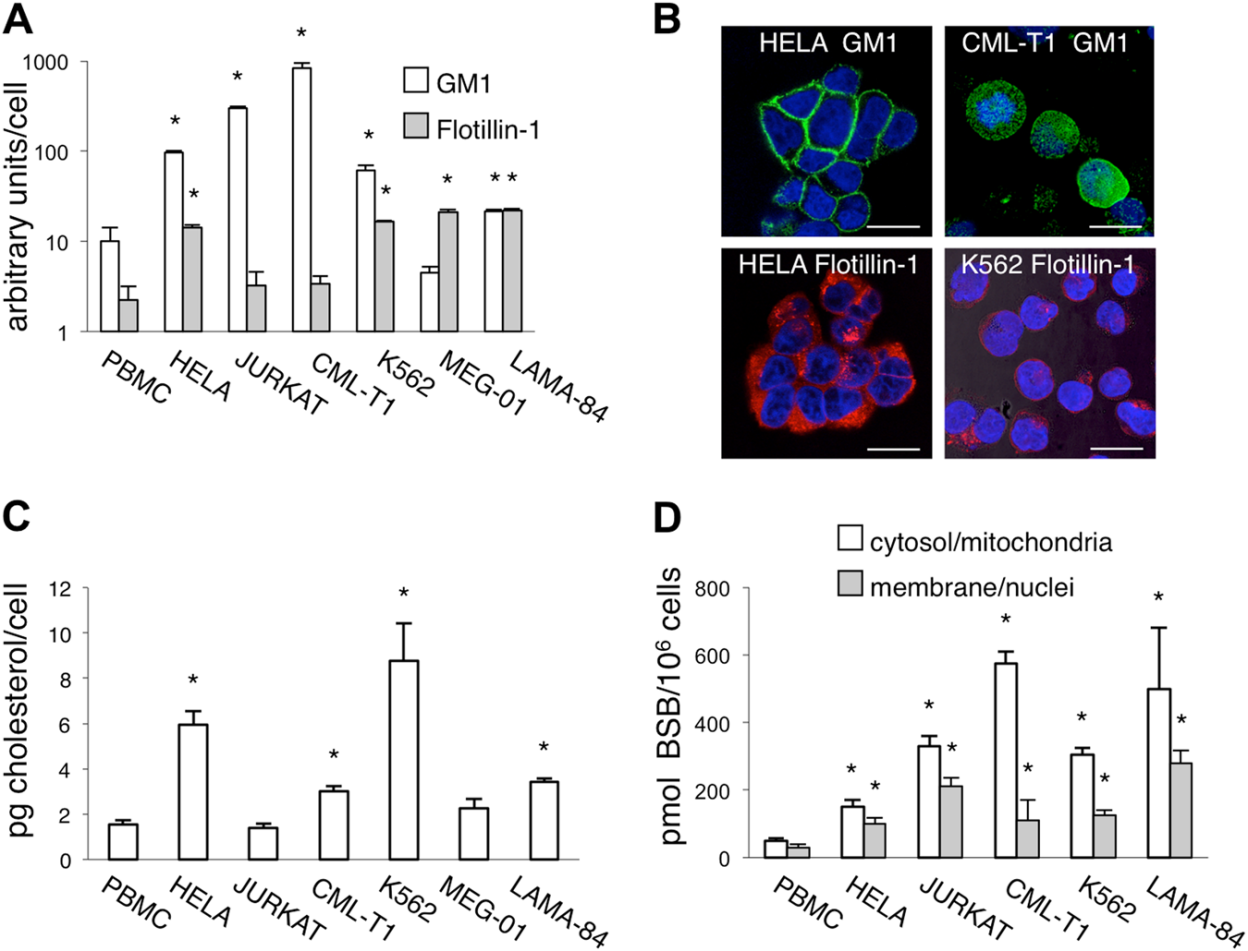
Lipid rafts and BSB uptake. **a** Expression of GM1 and flotillin-1 as assessed by flow cytometry in different cell types. Data were expressed as arbitrary units dividing the MFI by the median forward scatter (size) in each sample. *p < 0.05, as compared to PBMC. **b** Immunofluorescence detection of GM1 (Alexa Fluor 488) and Flotillin-1 (Cy-3) on different cell types. Samples were counterstained with DAPI and visualized by confocal microscopy. Magnification 63×. Scale bar: 17 μm. **c** Plasma membrane content of cholesterol. *p < 0.05 as compared to PBMC. **d** BSB concentrations in cellular compartments of different cells treated with 40 µM BSB for 24 h. *p < 0.05, as compared to PBMC. Mean values and s.d. of five independent determinations for each cell type were plotted.

It was previously suggested that BSB enter cells through lipid rafts, highly dynamic sterol- and sphingolipid-enriched membrane domains that function as platforms to concentrate signal transduction machineries. Ganglioside M1 (GM1), flotillin-1, and cholesterol are three of the main raft components and are widely used as markers for lipid raft cellular amount^10,12^. To test the possibility that BSB specificity for transformed cells may reside in different lipid raft contents as compared to the normal counterparts, we first quantified by flow cytometry GM1 and flotillin-1 in either peripheral blood mononuclear cells (PBMC) or a panel of cancer cell lines (Fig. 1a), some of them being also visualized by confocal microscopy (Fig. 1b). With respect to PBMC, most tumor cells had superior amounts of GM1 and flotillin-1, detected by means of the affinity to cholera toxin B and a specific monoclonal antibody, respectively. Cellular cholesterol content, determined by high-performance liquid chromatography (HPLC), also resulted on average significantly more abundant in neoplastic cells than PBMC (Fig. 1c). In parallel, we measured BSB content in subcellular fractions. As compared to PBMC, HeLa, Jurkat, CML-T1, K562, and LAMA-84 cancer cells internalized higher amounts of BSB, which accumulated in the cytosolic/mitochondrial fraction and, to a lesser extent, in cell membranes and nuclei (Fig. 1d). Therefore, lipid raft amount correlated with BSB intracellular content. In addition, it correlated with cell sensitivity to BSB, as evaluated by 3-(4,5-dimethylthiazol-2-yl)-2,5-diphenyltetrazolium bromide (MTT) assay and expressed as μM IC_50_ (half-maximal inhibitory concentration; Table 1). Our previous observation that filipin III, a polyene antibiotic disrupting cholesterol-rich raft fractions, rescued cells from BSB-induced cytotoxicity further strengthens the importance of membrane rafts in the BSB mechanism of action^6^.

**Table 1:** Relationship between lipid raft components (GM1, flotillin-1, and cholesterol) and sensitivity to BSB, as expressed by IC_50_ (μM) in PBMC and human cancer cell lines.

Cell line	GM1 (a)	Flotillin-1 (b)	Cholesterol (c)	a + b + c	IC_50_	[Cellular BSB]
PBMC	+	+	+	+++	181	70 ± 9
HeLa	+ +	+	+ +	+ ++++	70	250 ± 20
JURKAT	+ + +	+	+	+ ++++	80	541 ± 27
CML-T1	+ + +	+	+	+ ++++	53	669 ± 47
K562	+ +	+ +	+ +	+ + ++++	73	407 ± 17
MEG-01	+	+ +	+	+ + + +	63	nd
LAMA-84	+ +	++	+	+++++	68	765 ± 109

*nd* not done

GM1 and flotillin-1 were expressed as arbitrary units of fluorescence based on the results of fluorescence-activated cell sorting analysis. The ratio between mean fluorescence intensity (MFI) and mean forward scatter of the cells was determined (≤15 = +; 15–100 = + +; >100 = + + +), to correct for difference in cell sizes. Cholesterol was expressed as pg/cell (≤3 = +; >3 = + +). [BSB] was expressed as pmol/10^6^ cells

### Cytostatic and cytotoxic effects of BSB

**Fig. 2:**
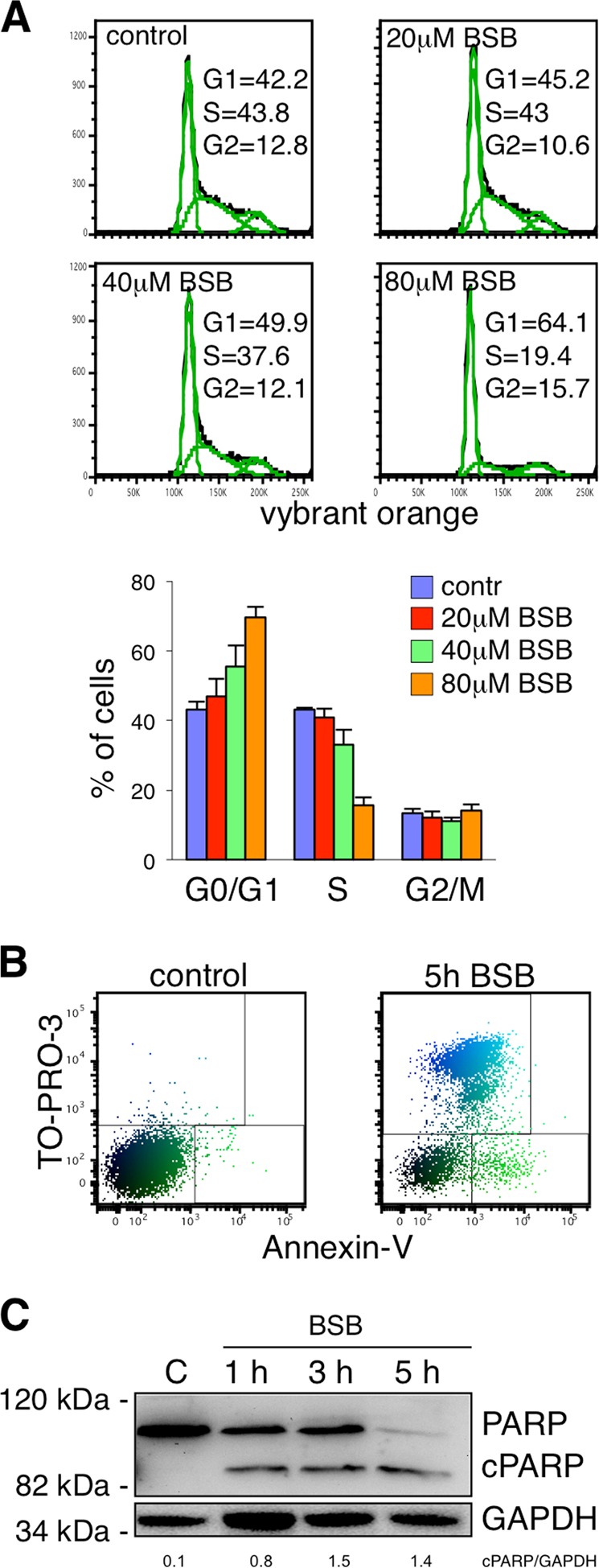
Cell cycle blockage and proapoptotic activity of BSB. **a** Top: a representative flow cytometric experiment demonstrating cell cycle arrest by BSB. Jurkat cells treated with 20, 40, and 80 µM BSB for 24 h were incubated with 10 µM Vybrant DyeCycle orange stain for 15 min. Bottom: statistical analysis (mean and s.d.) of the cell cycle phases. **b** Cytometric PolyChromatic plot analysis of apoptosis after 5-h treatment with 80 µM BSB. Jurkat cells were stained with Annexin-V and TO-PRO-3 to distinguish between alive (Annexin-V^−^/TO-PRO-3^−^), early apoptotic (Annexin-V^+^/TO-PRO-3^−^), and late apoptotic/necrotic cells (Annexin-V^+/−^/TO-PRO-3^+^). **c** Western blot showing the appearance of the cleaved form of PARP (cPARP) following 1, 3 and 5 h treatment with 80 μM BSB in Jurkat cells. Each experiment is representative of five.

We previously reported that BSB decreased viability of several neoplastic cell types^5,7,8,10^. Here we analyzed in more detail the cytostatic and cytotoxic effects of BSB in HeLa and Jurkat cells. Flow cytometric analysis of DNA content revealed that 24-h treatment with BSB arrested cells in G_0_/G_1_ phase in a concentration-dependent manner. At the same time, the number of S phase cells decreased (Fig. 2a). Annexin-V/TO-PRO-3 staining performed after 5-h BSB treatment showed an increase of both early apoptotic (Annexin-V^+^/TO-PRO-3^−^) and late apoptotic/necrotic cells (Annexin-V^+/−^/TO-PRO-3^+^; Fig. 2b). Exposure to 80 μM BSB also increased the level of the apoptotic marker cleaved poly(ADP-ribose) polymerase (PARP) in Jurkat cells (Fig. 2c; *p* < 0.05). These results indicated that BSB exerted a direct growth inhibitory effect on transformed cells that was mediated through cell cycle arrest in G_0_/G_1_ phase and induction of RCD.

### BSB-induced BID translocation to mitochondria and lysosomes

**Fig. 3:**
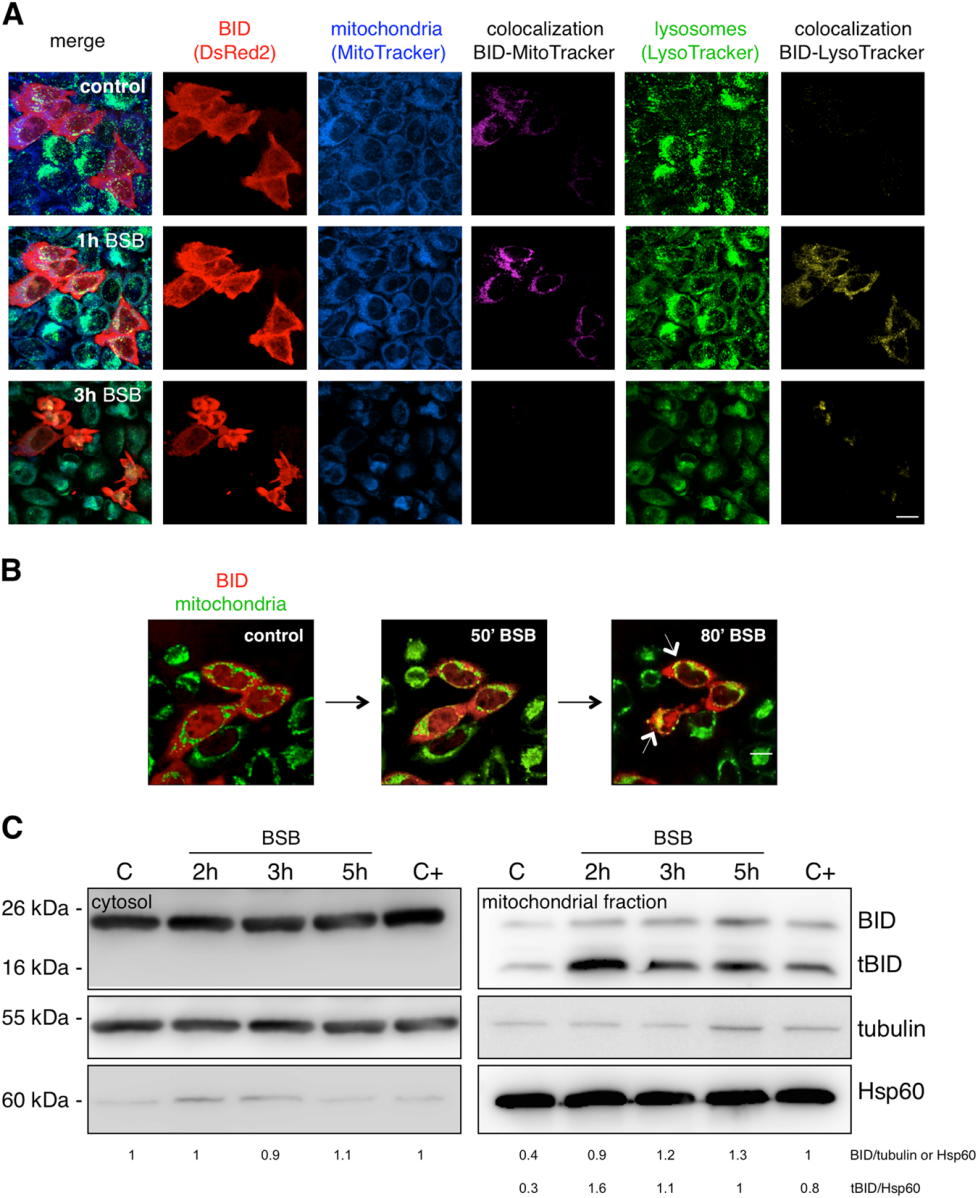
Fate of BID following BSB stimulation. **a** Colocalization of pDsRed2-BID with the specific mitochondrial (MitoTracker Deep Red FM) and lysosomal (LysoTracker green DND-26) dyes was analyzed by time-lapse imaging confocal microscopy. pDsRed2-BID-transfected HeLa cells were stained with the probes, stimulated with 80 μM BSB and monitored for 3 h. One hour after addition of BSB, the uniformly distributed red fluorescence in the cytosol, spatially distinct from the blue granular mitochondrial and the green granular lysosomal ones, appeared translocated and colocalized with mitochondria (purple) and lysosomes (yellow), as clearly appreciable at the level of the single optical slices. Three hours after addition of BSB, the apoptotic features were clear: the uniform red pattern was completely replaced by red clusters; blue and green fluorescences (and respective colocalizations) decreased as a consequence of dye leakage. Scale bar: 17 μm. **b** Key frames selected from Supplementary Movie 1. 80’ stimulation with BSB led to the overlap between green-stained mitochondria and red fluorescent BID, generating yellow signals (white arrows). Scale bar: 17 μm. **c** Kinetic western blot demonstrating the appearance of tBID and, to a lesser extent, full-length BID in the mitochondrial fraction. The positive control (C+) of BID cleavage is etoposide. The shown experiment is representative of three.

Upon BSB entry, a number of intracellular pathways involving apoptotic and autophagic mechanisms must be triggered out to provoke the cell death we observed. Based on our previous biochemical studies, demonstrating that BSB can recruit and directly interact with BID within lipid rafts^10^, we focused on this BH3-only protein and analyzed its subcellular fate upon BSB stimulation. HeLa cells were transfected with pDsRed2-BID expression vector, whose product was monitored by confocal microscopy to assess its redistribution through the intracellular compartments. As soon as 1 h after BSB stimulation, BID translocated to mitochondria and lysosomes, as evidenced by its tight colocalization with MitoTracker and LysoTracker fluorescent dyes, respectively (Fig. 3a, b and Supplementary Movie 1). This was followed by organelle damage, as indicated by the loss of fluorescence intensity of mitochondria and lysosomes, which started as soon as 3 h after BSB stimulation (Fig. 3a). Kinetic immunoblot analysis demonstrated that both full-length and truncated BID (tBID) were present in mitochondrial extracts of BSB-treated cells (Fig. 3c; *p* < 0.05), further supporting its subcellular relocation upon BSB exposure. Thus, the cell death executioner organelles, mitochondria and lysosomes, are targets of BID being mobilized by BSB.

### Autophagic flux impairment and lysosome destabilization

The lysosomal compartment, one of the main targets of BSB, is critically involved in the autophagic pathway, a regulated self-digestion process triggered by unfavorable conditions, in which selected cytoplasmic material is sequestered into double-membrane vesicles (autophagosomes) and delivered for lysosomal degradation. Two fundamental components contribute to the autophagic flux: (a) autophagosome nucleation and maturation, a microtubule-associated protein 1 light chain 3 (LC3) and Beclin 1-dependent process; (b) functional lysosomes, eventually fusing with autophagosomes to efficiently degrade the autophagic cargo^22^.

**Fig. 4:**
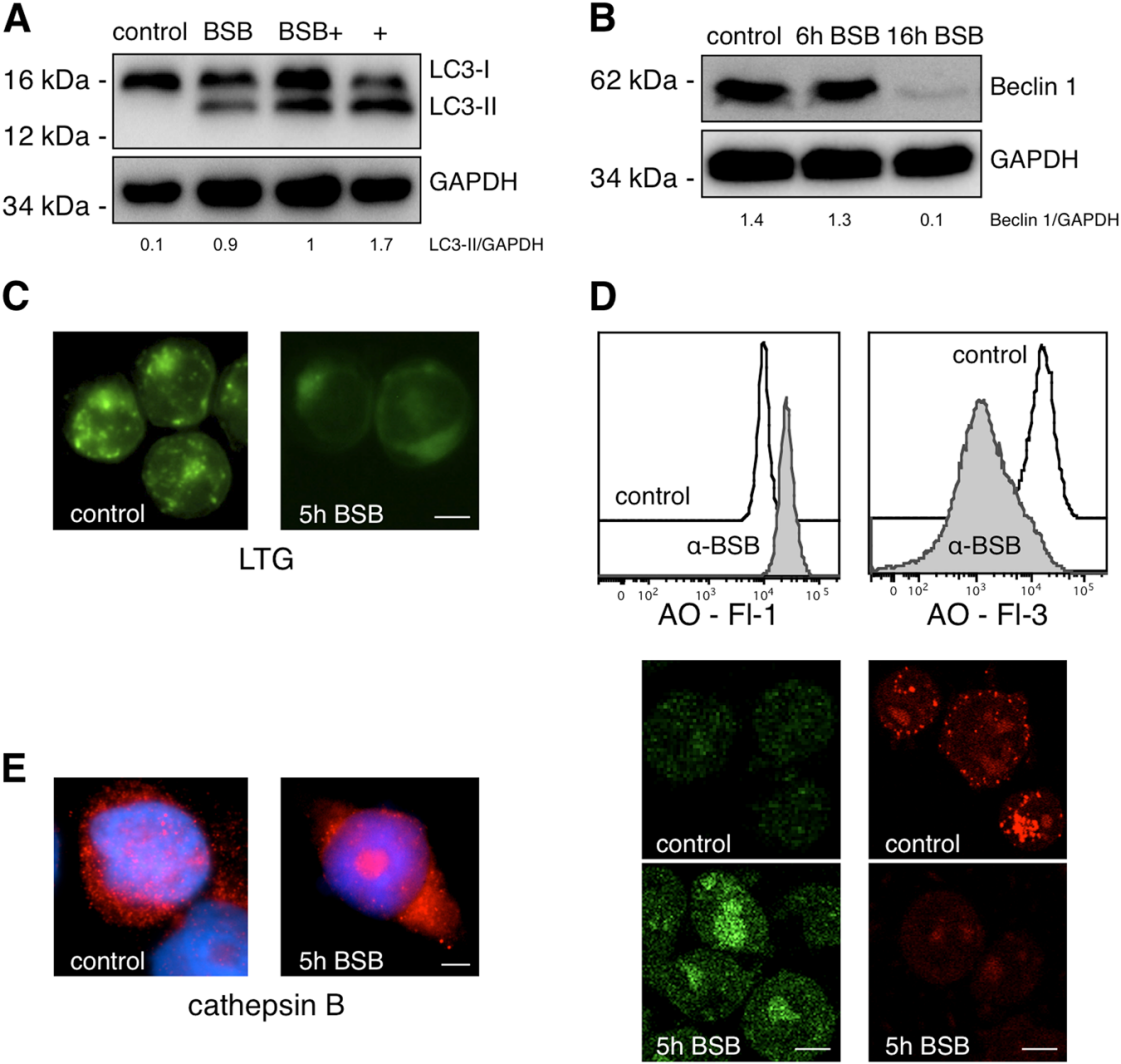
Autophagy impairment and lysosomal collapse induced by BSB. **a** Expression of LC3-I and LC3-II in Jurkat cells left untreated (control) or treated for 16 h with BSB, BSB plus pepstatin A and E-64D (BSB+), or pepstatin A and E64D without BSB (+). **b** Expression of Beclin 1 in Jurkat cells treated or not with BSB for 6 and 16 h. **c** Fluorescence microscopy of lysosomal destabilization evaluated by LTG-uptake assay in Jurkat cells. Shown is the shift from a punctate staining (intact lysosomes from untreated cells) to a diffuse cytoplasmic signal (disrupted lysosomes from treated cells). Scale bar: 12 μm. **d** Flow cytometry and fluorescence microscopy assessment of lysosomal membrane permeabilization. *AO-relocation assay (left)*: Jurkat cells stained with the metachromatic fluorophore AO were exposed to 80 µM BSB for 5 h. Leakage of lysosomal content into the cytoplasm was measured as an increase in green fluorescence (control 11,590 ± 1243 vs treated 17,185 ± 2532, *p* < 0.05). *AO-uptake assay (right)*: Cells treated with 80 μM BSB for 5 h were stained with AO. Reduced lysosomal AO accumulation was measured as a reduction in red fluorescence (control 15,290 ± 2379 vs treated 1600 ± 200, *p* < 0.001). Scale bar: 12 μm. **e** Fluorescence microscopy evaluation of the lysosomal protease cathepsin B after 5-h treatment of Jurkat cells with 80 µM BSB. Cathepsin B was released into the cytosol upon BSB stimulation, as evidenced by the diffuse fluorescence pattern. A large cytoplasmic cathepsin B aggregate was also evident. Samples were counterstained with DAPI. Scale bar: 12 μm.

(a) By means of Cyto-ID-Green staining, we have previously shown that BSB increased autophagosome formation in a dose-dependent manner. This may indicate either induction of autophagic flux or accumulation of autophagosomes due to a downstream blockage^6^. To clarify this aspect, we assessed the turnover of LC3-II, a widely used marker for autophagosome formation resulting from the lipidation of LC3-I, in the presence or absence of two lysosomal degradation inhibitors, E-64D and pepstatin A. In E-64D and pepstatin A-treated cells, an autophagy promoter will increase LC3-II levels, whereas an autophagy inhibitor will not change them^23^. Stimulation with BSB increased LC3-II amount as compared to control cells (*p* < 0.05). However, pretreatment with E-64D and pepstatin A did not further augment LC3-II levels (Fig. 4a; *p* = ns (not significant) between BSB and BSB plus autophagy inhibitors), demonstrating BSB-dependent impairment of autophagic flux. Of note, expression of Beclin 1 protein, which is dispensable for LC3-II lipidation but required for the subsequent autophagosome maturation^24^, was clearly reduced after 16-h treatment with BSB (Fig. 4b; *p* < 0.05). Conversely, Beclin 1 messenger RNA, evaluated by quantitative real‐time polymerase chain reaction (RT-qPCR), significantly increased upon BSB exposure both in HeLa and Jurkat cells (fold change of 4.3 ± 0.9 and 3 ± 0.2 at 16 h, respectively, *p* < 0.05, not shown), suggesting enhanced Beclin 1 protein degradation rather than transcriptional suppression.

Therefore, BSB led to the accumulation of Beclin 1-deficient, poorly progressing autophagosomes.

(b) By staining cells with LysoTracker green (LTG), we noticed lysosome accumulation as soon as 1 h after BSB administration (Fig. 3a), further indicating an altered autophagic process. Afterward, LTG uptake progressively decreased (Figs. 3a and 4c). This was compatible with an enlargement/accumulation and subsequent collapse of lysosomal compartment. To evaluate the effects of BSB on lysosomal membrane stability, we used the fluorescent lysosomotropic dye acridine orange (AO). The AO-relocation assay, aimed at the assessment of AO leakage outside the lysosome^25^, showed an increase in cytosolic green fluorescence after treatment with BSB (Fig. 4d; left). Accordingly, the AO-uptake assay, directly measuring the AO accumulation inside the lysosome, demonstrated a decrease in lysosomal red fluorescence (Fig. 4d; right). To further confirm the BSB-triggered loss of lysosomal membrane integrity, we stained cells with a fluorophore-labeled antibody detecting cathepsin B, a cysteine protease involved in enzymatic digestion of lysosomal content. A 5-h exposure to BSB determined lysosome-to-cytosol redistribution of cathepsin B, as evidenced by the diffuse fluorescence pattern together with the presence of large cytoplasmic cathepsin B aggregates (Fig. 4e).

Overall, BSB-induced Beclin 1 decrease and lysosomal disruption hindered the autophagic flux, which was likely impeded at the stage of autophagosome. The collapse of autophagic machinery eventually favored the leakage of lysosomal proapoptotic cathepsins throughout the cytosol.

### Trigger for mitochondrial damage

**Fig. 5:**
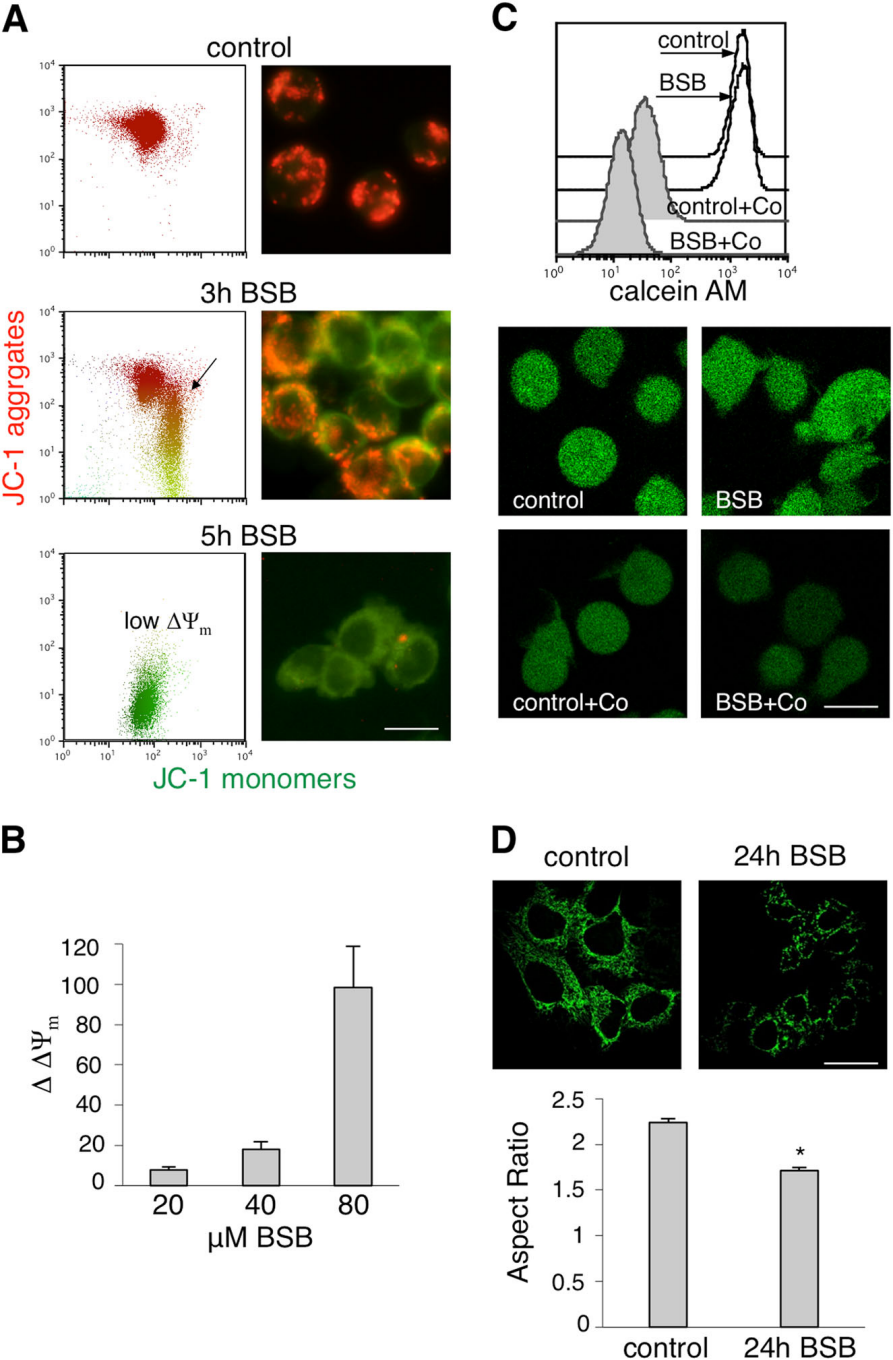
Mitochondrial damage triggered by BSB. **a** JC-1 technique demonstrating ΔΨ_m_ dissipation caused by BSB in Jurkat cells. *Flow cytometry (left, polychromatic plot analysis)*: The normal (high) ΔΨ_m_ of untreated cells moved downward (intermediate and low ΔΨ_m_) after treatment with 40 μM BSB, at the indicated time points. This was due to the progressive JC-1 dislocation from mitochondria to the cytosol. *Fluorescence microscopy (right)*: In untreated cells, well-polarized mitochondria appeared as a red punctate fluorescence; after treatment with 40 μM BSB red fluorescence was lowered and the green one was increased, indicating loss of ΔΨ_m_. Scale bar: 12 μm. **b** ΔΔΨm values, as evaluated by TMRM staining, of Jurkat cells exposed to 20, 40, and 80 µM BSB for 5 h were 7.6 ± 1.5, 18 ± 4, and 98.3 ± 20.6, respectively, as compared to untreated ones, *p* < 0.05. **c** Impairment of mPTP function after 5-h treatment with 40 μM BSB, as assessed by calcein AM assay in Jurkat cells. The fluorescence intensity of calcein AM-loaded cells was similar between resting and treated ones (1554 ± 120 vs 1568 ± 115). Quencing of calcein AM fluorescence by CoCl_2_ (Co) was significantly reduced in treated cells as compared to control (18 ± 4 vs 34 ± 3, *p* < 0.05), indicating mPTP opening. Scale bar: 12 μm. **d** HeLa cells transfected with mito-GFP were treated with 80 µM BSB or vehicle for 24 h and mitochondrial morphology was monitored. Upper panel: representative images of mitochondrial network by live cell imaging. Scale bar: 10 µm. Lower panel: aspect ratio analysis represented as histogram with s.e.m. Data indicate significant fragmentation of mitochondrial network upon BSB exposure. A total of 500 measurements for each condition have been made. *p < 0.001. For panels **a**, **c**, and **d**, shown experiment is representative of at least five.

BID translocation to mitochondria (Fig. 3a–c) was expected to initiate the intrinsic apoptotic pathway through mitochondrial membrane permeabilization (MMP), considered as the point of no return toward cell death^26^. We measured MMP by means of JC-1, a mitochondrial transmembrane potential (ΔΨm)-sensitive dye, which accumulates in well-polarized mitochondria in form of red fluorescent aggregates^8^. Figure 5a shows that treatment with 40 μM BSB resulted in a time-dependent decrease of JC-1 red fluorescence, shifting downward due to ΔΨm dissipation. This was further confirmed by using tetramethylrhodamine methyl ester (TMRM), a potentiometric fluorescent probe accumulated by mitochondria in a log scale as ΔΨm increases, according to Nernst equation in a voltage-dependent manner^27^. Considering 180 mV as control cell ΔΨm value^27^, we calculated a significant fall to 172 ± 1.5, 162 ± 4, and 81 ± 20 mV in cells treated with 20, 40, and 80 µM BSB, respectively. As such, there was a dose-dependent increase of ΔΔΨm (Fig. 5b), defined as the difference between control cell- and treated cell-ΔΨm.

BSB-induced MMP may also result from the opening of mitochondrial permeability transition pore (mPTP). To explore this possibility, the calcein acetoxymethyl ester (calcein AM) assay was performed. Calcein AM becomes fluorescent in cells after cleavage of AM groups through cytosolic and mitochondrial esterases. CoCl_2_ cannot enter mitochondria when mPTP is closed, so that it quenches cytosolic fluorescence leaving unmodified the mitochondrial one^7^. Control and BSB-treated cells showed similar MFI when loaded with only calcein AM. However, CoCl_2_ addition led to a far more MFI decrease in treated cells as compared to control due to the quenching of both cytosolic and mitochondrial fluorescence (Fig. 5c), reflecting the irreversible mPTP opening.

Mitochondrial membrane depolarization induced by BSB could associate with altered mitochondrial morphology^28^. To investigate this point, HeLa cells were transfected with mitochondrial-targeted green fluorescent protein (GFP) and mitochondrial morphology was assessed in live cells by confocal microscopy. BSB-treated cells displayed more discrete, less interconnected, mitochondria as compared to control. In particular, aspect ratio (defined as the ratio between the major axis and minor axis of each particle) decreased after BSB exposure, clearly indicating mitochondrial network fragmentation (Fig. 5d).

These findings suggested that BSB perturbed mitochondrial membranes and dissipated ΔΨm, two well-recognized events precipitating, or at least amplifying, RCD pathways^14^.

### BID knockdown protects cancer cells from BSB-induced cytotoxicity

**Fig. 6:**
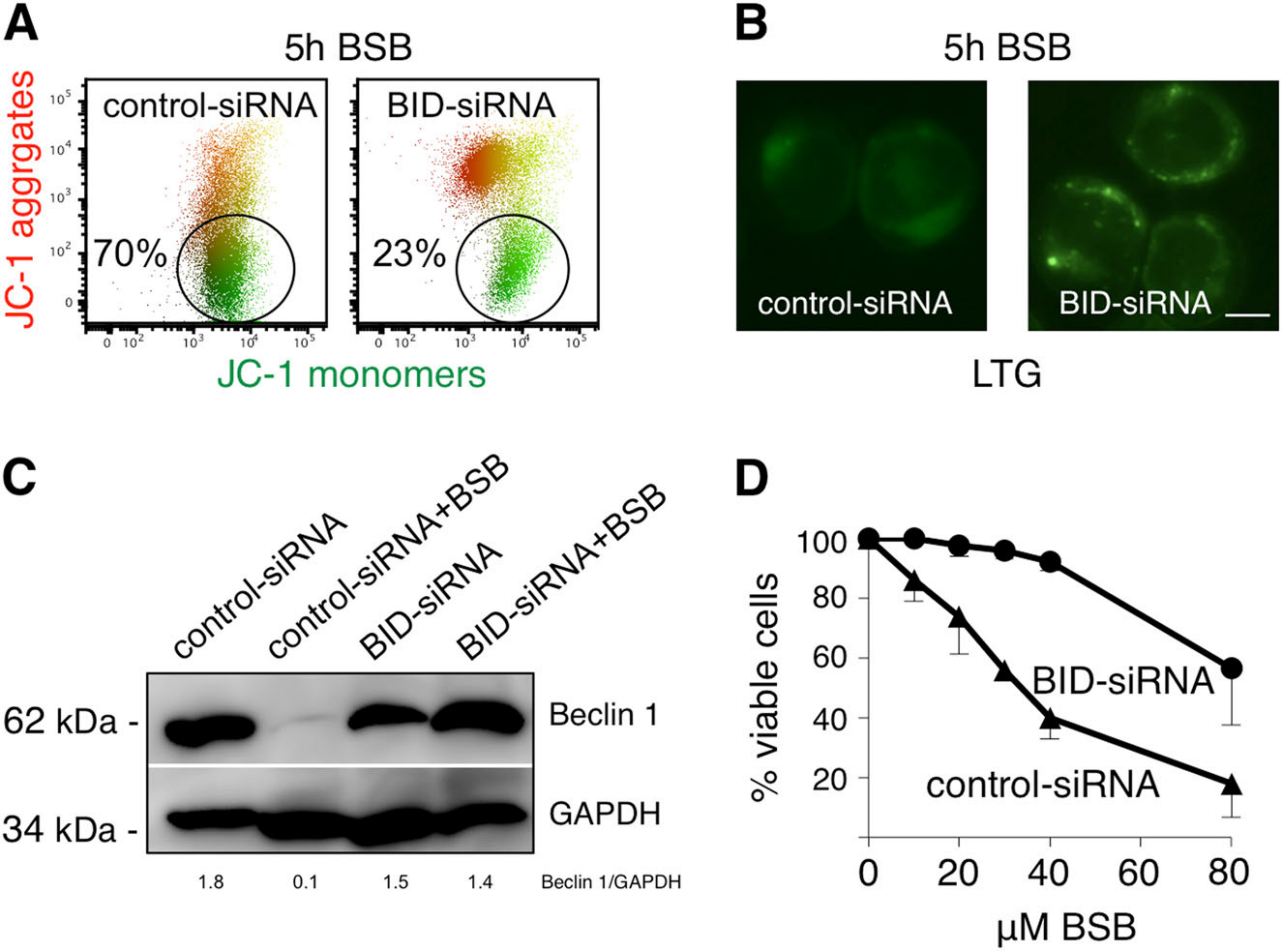
Rescuing effects of BID knockdown on BSB-treated HeLa cells. **a** ΔΨm dissipation after 5-h treatment with 40 μM BSB as assessed by JC-1 staining in flow cytometry. The decrease of JC-1 red fluorescence, typical of cells with depolarized mitochondria, is significantly more evident in control as compared to BID-knockdown cells. **b** Lysosomal destabilization after 5-h treatment with 40 μM BSB as evaluated by LTG-uptake assay. Shown is the shift from weak and diffuse cytoplasmic signal (disrupted lysosomes from control-siRNA cells) to a punctate pattern (intact lysosomes from BID-siRNA cells). Scale bar: 12 μm. **c** Expression of Beclin 1 in control and BID-knockdown cells after 16-h treatment with 80 μM BSB. Shown experiments are representative of 3. **d** Viability as assessed by MTT assay in cells transfected with control-siRNA or BID-siRNA and exposed to 10, 20, 30, 40, and 80 µM BSB for 96 h. Mean values and s.d. of six independent determinations were plotted.

To verify the actual contribution of BID in the cytotoxic program of BSB, we generated BID-knockdown HeLa cells and analyzed organelle damage after BSB exposure. BID silencing partially protected cancer cells from BSB-induced mitochondrial depolarization, as evidenced by the decreased percentage of green-emitting JC-1 monomers compared to control (Fig. 6a). In addition, BSB-triggered lysosomal membrane permeabilization was attenuated in BID-knockdown cells, still showing some LTG puncta after 5-h treatment (Fig. 6b). Beclin 1 suppression, clearly evident after 16-h exposure to BSB, was significantly prevented by BID silencing (Fig. 6c; *p* < 0.05). Thus, BID may be directly involved in autophagy downregulation and BID-knockdown cells may hold functional autophagic machinery, potentially rescuing cells from apoptosis. Indeed, we found that at concentrations up to 80 μM, which were usually associated with an incremental degree of cell death, BID-silenced cells were significantly protected from BSB cytotoxicity (Fig. 6d). This makes BID a proapoptotic and anti-autophagic molecular player, whose activity is necessary to achieve the full cell death program of BSB.

## Discussion

In the search of novel therapeutic approaches against cancer, synthetic molecules selectively targeting tumor surface receptors, oncogenic kinases, or immune checkpoints largely predominate, with remarkable results^29–31^. However, they usually fail to eradicate the neoplastic clone. Even in chronic myeloid leukemia, perhaps representing the clearest example of tyrosine kinase inhibitor efficacy, the neoplastic stem cell compartment usually survives due to its basic autophagic, cell-rescuing, mechanisms^32^. On the other side, plant-derived compounds have notoriously been considered as dirty molecules, given their propensity to affect numerous intracellular pathways^2^. But they should be of interest to cancer treatment due precisely to their ability to modulate the eukaryotic autophagic and apoptotic machineries, two intersected and evolutionarily conserved programs often dysregulated in cancer cells.

Our study provides evidence that the natural lipophilic terpene BSB arrests cell cycle and induces neoplastic cell death through a BID-dependent mechanism. In particular, BSB (a) selectively accumulated in transformed cells, (b) triggered BID translocation to lysosomes and mitochondria, (c) permeabilized lysosomal and mitochondrial membranes leading to cytoplasmic leakage of apoptogenic factors, as well as bioenergy dissipation, and (d) blocked the autophagic flux, possibly lowering the threshold for cell death initiation.

Different cell-free assays have previously shown that direct interactions between BSB and the non-BH3 domain of BID are biochemically possible^10^. In addition, lipids act as ligands for BID, which is also devoted to their transport between intracellular membranes^15^. The subcellular localization in turn regulates the proapoptotic functions of the BCL-2 family members^33^. At resting conditions, BID is anchored to the plasma membrane or free floating in the cytoplasm. BSB rapidly increased BID affinity for specific intracellular membranes, favoring its mobilization to lysosomes and mitochondria (Fig. 3a, b). This was meant to have a great impact on the available binding partners, creating an organelle-centered imbalance between prosurvival and proapoptotic factors in favor of the latter. Together with BIM, BID is known to activate the proapoptotic effectors BAK and BAX, eventually forming oligomers with membrane-permeabilizing properties^34^. In our setting, both full-length and, to a major extent, tBID were found in the mitochondrial fraction upon BSB stimulation (Fig. 3c). Thus, BSB binding to BID may impose specific sublocalizations and increased sensitivity to enzymatic cleavage as well. Alternatively, very early and still undefined molecular events may link BSB entry to BID cleavage. In this regard, the upregulation of specific death receptors^35^ is one possibility we are currently testing.

The irreversible damage of lysosomes and mitochondria had an impact on autophagic and apoptotic pathways. Despite a transient autophagy induction^6^, representing the cell’s attempt to survive, BSB substantially impaired the autophagic flux through downmodulation of Beclin 1, which is required for autophagosome maturation^24^, and disruption of lysosomal compartment (Fig. 4a–e). AO relocation to the cytosol, cathepsin B leakage, and decreased LysoTracker uptake well documented the lysosomal membrane permeabilization and the loss of acidic environment typical of lysosomes. This appears to be mediated, at least in part, by BID activation because lysosomal membrane of BID-knockdown cells partially preserved its integrity (Fig. 6b). In addition, BID silencing completely prevented BSB-induced Beclin 1 suppression (Fig. 6c). These findings are consistent with a previous paper where BID was hypothesized to act as a molecular switch between apoptosis and autophagy^36^. When BID is expressed and activated by BSB, Beclin 1 ends up being degraded and autophagy collapses; when BID is not available, Beclin 1 persists and the continuous autophagic flux probably helps cancer cells survive. Further investigations about BID/Beclin 1 interplay may uncover novel non-apoptotic functions of BID that are potentially useful for therapeutic purposes.

Our data show that mitochondrial mechanisms belonging to intrinsic apoptosis play a role in BSB-induced cell death. Mitochondria became dysfunctional under BSB (Fig. 5a–d): their outer membrane was permeabilized, the mPTP opened, and the transmembrane potential progressively declined, an irreversible cascade associated with organelle fragmentation. The analysis of BID fate, moving to mitochondria and lysosomes at the same time, suggested that intrinsic apoptotic pathway and lysosomal cell death might be triggered in parallel. Therefore, both mitochondrial and lysosomal death subroutines are likely to concur to BSB-induced cell suicide^14^. This is consistent with our previous observation that broad inhibitors of caspases, the very downstream effectors of mitochondrial apoptosis, did not confer substantial cytoprotection. In contrast, pretreatment with small molecules that abolished the early autophagy attempts further unleashed cell death machineries^6^. As fragmented mitochondria need to be degraded by functional lysosomes to maintain organelle homeostasis^37^ and avoid persistent exposure of BID, it is tempting to hypothesize a self-amplifying mitochondrial–lysosomal death loop promoted by BSB, eventually turning autophagy into a destructive process. A similar mechanism has already been proposed for the pentacyclic triterpenoid betulinic acid^38^.

Why BSB cytotoxic action preferentially targets cancer cells over their non-neoplastic counterparts is still an unanswered point. As shown in Fig. 1d, neoplastic cell lines took up higher amount of BSB than PBMC, possibly reaching the threshold concentration that triggers death cascade. Lipid rafts, which are far more represented in transformed cells^12^, positively correlated with intracellular BSB concentrations and may act as a preferential entry route, conferring some degree of cancer selectivity. However, other aspects of basic cancer biology must be involved. Transformed cells are often primed for death, as death signaling from oncogenic lesions causes anti-apoptotic BCL2 family members to sequester high quantities of proapoptotic BH3 proteins. In other words, tumor cell mitochondria are addicted to their anti-apoptotic arsenal to prevent MMP^39^. In this context, massive BSB-induced BID translocation would disengage the activator BIM from its binding partners or directly activate the effectors BAK/BAX, successfully overwhelming tumor anti-apoptotic reserves. More generally speaking, the death priming together with a peculiar genetic reprogramming^40^ might determine the outcome of xenohormesis in cancer cells. Perhaps, dirtiness of plant-derived molecules^2^ turns into clinical advantage when multiple cell death subroutines need to be activated simultaneously.

In conclusion, we provide evidence that the cytotoxic effect of BSB passes through BID, which has a nonredundant role in cell death initiation. Parallel damage of lysosomes and mitochondria, which ends up causing autophagy collapse and RCD, characterizes the mode of action of BSB. Given its preclinical efficacy, low general toxicity, and affordable cost, we propose BSB as a good candidate for future anticancer strategies.

## Supplementary information

Supplementary movie 1

## References

[1] Rodrigues FFG (2018). In vitro antimicrobial activity of the essential oil from Vanillosmopsis arborea Barker (Asteraceae) and its major constituent, α-bisabolol. Micro. Pathog..

[2] Howitz KT, Sinclair DA (2008). Xenohormesis: sensing the chemical cues of other species. Cell.

[3] Meeran MFN, Laham F, Al-Taee H, Azimullah S, Ojha S (2018). Protective effects of α-bisabolol on altered hemodynamics, lipid peroxidation, and nonenzymatic antioxidants in isoproterenol-induced myocardial infarction: In vivo and in vitro evidences. J. Biochem Mol. Toxicol..

[4] Braga PC, Dal Sasso M, Fonti E, Culici M (2009). Antioxidant activity of bisabolol: inhibitory effects on chemiluminescence of human neutrophil bursts and cell-free systems. Pharmacology.

[5] Bonifacio M (2012). α-Bisabolol is an effective proapoptotic agent against BCR-ABL(+) cells in synergism with Imatinib and Nilotinib. PLoS ONE.

[6] Rigo A, Vinante F (2016). The antineoplastic agent α-bisabolol promotes cell death by inducing pores in mitochondria and lysosomes. Apoptosis.

[7] Rigo A (2018). Efficient lysis of B-chronic lymphocytic leukemia cells by the plant-derived sesquiterpene alcohol α-bisabolol, a dual proapoptotic and antiautophagic agent. Oncotarget.

[8] Cavalieri E (2011). Pro-apoptotic activity of α-bisabolol in preclinical models of primary human acute leukemia cells. J. Transl. Med..

[9] Cavalieri E (2004). alpha-Bisabolol, a nontoxic natural compound, strongly induces apoptosis in glioma cells. Biochem. Biophys. Res. Commun..

[10] Darra E (2008). Insight into the apoptosis-inducing action of alpha-bisabolol towards malignant tumor cells: involvement of lipid rafts and Bid. Arch. Biochem. Biophys..

[11] Murata Y (2017). The anticancer effects of novel α-bisabolol derivatives against pancreatic cancer. Anticancer Res..

[12] Patra SK (2008). Dissecting lipid raft facilitated cell signaling pathways in cancer. Biochim. Biophys. Acta.

[13] Sandra F (2005). Tumor necrosis factor-related apoptosis-inducing ligand alters mitochondrial membrane lipids. Cancer Res..

[14] Galluzzi L (2018). Molecular mechanisms of cell death: recommendations of the Nomenclature Committee on Cell Death 2018. Cell Death Differ..

[15] Degli Esposti M, Erler JT, Hickman JA, Dive C (2001). Bid, a widely expressed proapoptotic protein of the Bcl-2 family, displays lipid transfer activity. Mol. Cell Biol..

[16] Contreras JA, Castro M, Bocos C, Herrera E, Lasuncion MA (1992). Combination of an enzymatic method and HPLC for the quantitation of cholesterol in cultured cells. J. Lipid Res..

[17] Imai H (1998). Suppression of leukotriene formation in RBL-2H3 cells that overexpressed phospholipid hydroperoxide glutathione peroxidase. J. Biol. Chem..

[18] São Pedro A, Detoni C, Ferreira D, Cabral-Albuquerque E, Sarmento B (2009). Validation of a high-performance liquid chromatography method for the determination of (-)-alpha-bisabolol from particulate systems. Biomed. Chromatogr..

[19] Carcereri de Prati A (2017). Metastatic breast cancer cells enter into dormant state and express cancer stem cells phenotype under chronic hypoxia. J. Cell Biochem..

[20] Rigo A (2012). CXCL12 and [N33A]CXCL12 in 5637 and HeLa cells: regulating HER1 phosphorylation via calmodulin/calcineurin. PLoS ONE.

[21] Marchi S, Bonora M, Patergnani S, Giorgi C, Pinton P (2017). Methods to assess mitochondrial morphology in mammalian cells mounting autophagic or mitophagic responses. Methods Enzymol..

[22] Mariño G, Niso-santano M, Baehrecke EH, Kroemer G (2014). Self-consumption: the interplay of autophagy and apoptosis. Nat. Rev. Mol. Cell Biol..

[23] Klionsky DJ (2016). Guidelines for the use and interpretation of assays for monitoring autophagy (3rd edition). Autophagy.

[24] He R, Peng J, Yuan P, Xu F, Wei W (2015). Divergent roles of BECN1 in LC3 lipidation and autophagosomal function. Autophagy.

[25] Servais H (2005). Gentamicin-induced apoptosis in LLC-PK1 cells: involvement of lysosomes and mitochondria. Toxicol. Appl. Pharm..

[26] Tait, S. W. G. & Green, D. R. Mitochondrial regulation of cell death. *Cold Spring Harb. Perspect. Biol.* **5**, pii:a008706 (2013). 10.1101/cshperspect.a008706 PMC3753705 24003207

[27] Galluzzi L (2007). Methods for the assessment of mitochondrial membrane permeabilization in apoptosis. Apoptosis.

[28] Archer SL (2013). Mitochondrial dynamics-mitochondrial fission and fusion in human diseases. N. Engl. J. Med..

[29] Lokhorst HM (2015). Targeting CD38 with daratumumab monotherapy in multiple myeloma. N. Engl. J. Med..

[30] Byrd JC (2013). Targeting BTK with ibrutinib in relapsed chronic lymphocytic leukemia. N. Engl. J. Med..

[31] Ansell SM (2014). PD-1 blockade with nivolumab in relapsed or refractory hodgkin’s lymphoma. N. Engl. J. Med..

[32] Kuntz EM (2017). Targeting mitochondrial oxidative phosphorylation eradicates therapy-resistant chronic myeloid leukemia stem cells. Nat. Med..

[33] Kale J, Osterlund EJ, Andrews DW (2018). BCL-2 family proteins: changing partners in the dance towards death. Cell Death Differ..

[34] Sarosiek KA (2013). BID preferentially activates BAK while BIM preferentially activates BAX, affecting chemotherapy response. Mol. Cell.

[35] Jin M (2019). Possible involvement of Fas/FasL-dependent apoptotic pathway in α-bisabolol induced cardiotoxicity in zebrafish embryos. Chemosphere.

[36] Lamparska-Przybysz M, Gajkowska B, Motyl T (2005). Cathepsins and BID are involved in the molecular switch between apoptosis and autophagy in breast cancer MCF-7 cells exposed to camptothecin. J. Physiol. Pharm..

[37] Youle RJ, Narendra DP (2011). Mechanisms of mitophagy. Nat. Rev. Mol. Cell Biol..

[38] Martins WK (2015). Parallel damage in mitochondrial and lysosomal compartments promotes efficient cell death with autophagy: the case of the pentacyclic triterpenoids. Sci. Rep..

[39] Certo M (2006). Mitochondria primed by death signals determine cellular addiction to antiapoptotic BCL-2 family members. Cancer Cell.

[40] Trigos AS, Pearson RB, Papenfuss AT, Goode DL (2017). Altered interactions between unicellular and multicellular genes drive hallmarks of transformation in a diverse range of solid tumors. Proc. Natl Acad. Sci. USA.

